# Effects of *Vaccinium* Berries on Serum Lipids: A Meta-Analysis of Randomized Controlled Trials

**DOI:** 10.1155/2015/790329

**Published:** 2015-08-09

**Authors:** Yitong Zhu, Ya Miao, Zheying Meng, Yuan Zhong

**Affiliations:** ^1^Department of Geriatrics, Shanghai Jiao Tong University Affiliated Sixth People's Hospital, No. 600 Yishan Road, Shanghai 200233, China; ^2^Department of Ultrasound in Medicine, Shanghai Jiao Tong University Affiliated Sixth People's Hospital, Shanghai Institute of Ultrasound in Medicine, No. 600 Yishan Road, Shanghai 200233, China

## Abstract

The beneficial effects of anthocyanins consumption on cardiovascular risk are supported by mechanistic and epidemiologic evidence. In order to explore the effects of *Vaccinium* berries rich in anthocyanins on serum lipids, we conducted a meta-analysis of relevant randomized controlled trials (RCTs). Sixteen studies with 1109 subjects were included in this meta-analysis. Significant heterogeneity confirmed differential effects between *Vaccinium* subclasses. The whortleberry group is significantly superior to placebo in lipids improvement. Besides, bilberry groups show significant differences in reducing LDL-C and increasing HDL-C in comparison with other treatments. For many of the other subgroups and comparison arms, there was insufficient evidence to draw conclusions about efficacy.

## 1. Introduction

Cardiovascular diseases, especially coronary heart disease (CHD) and stroke, are the leading causes of death worldwide [1]. One of the known risk factors for CHD is elevated serum lipid [2, 3]. Previous studies suggest that increasing high density lipoprotein cholesterol (HDL-C) and reducing triglycerides and small low density lipoprotein cholesterol (LDL-C) particles may have positive impact in prevention of CHD [4]. Current guidelines support LDL-C as a primary target of therapy [5]. General recommendation for lowering elevated lipid is developing a healthy lifestyle, including quitting smoking, exercising regularly, and low-oil, low-salt, low-fat diet. In reality, few people can strictly follow the above requirements, so drug therapy is of great necessity. As we know, statins are the first choice prescribed to achieve the goal of lipid-lowering effect [6]. Considering residual cardiovascular risk that remains after statin therapy, such as declines in hepatic function [7], muscle toxicity [8], and increasing risks of diabetes [9, 10], there is a strong demand for novel lipid-modifying agents that can be easily implemented by the majority of the population. The substitute should be safe without any toxic or side effect and rich in nutrients and have a prevention effect on hyperlipidemia. One of the promising alternatives is* Vaccinium* berry.

*Vaccinium* is a genus of shrubs or dwarf shrubs in the plant family Ericaceae. The fruits of many species are eaten by humans and some are of medicinal value, including cranberry, blueberry, bilberry, and whortleberry. The common characteristic of the* Vaccinium* berries is the abundant polyphenols content [11, 12], such as flavonols, phenolic acids, and anthocyanins. Anthocyanins have been reported to have a positive impact on inflammation, hypertension, hyperglycemia, oxidative damage, obesity, and lipid metabolism disorders [13–20]. In recent years, human and animal experiments have gradually found the lipids-lowering effects of extracts from different plants rich in anthocyanins [15, 21–25]. However it is still controversial, because the results of reported randomized controlled trials (RCTs) appear contradictory. Besides, different species of plants may have different effects on lipids metabolism. We cannot conclude which source of anthocyanins is having the most significant effect. In order to make clear the effect of* Vaccinium* berries, we conduct a meta-analysis of randomized controlled trials. We also selected* Vaccinium* berries based on the fact that these berries are commercially available all over the world, and therefore, our study findings may have guiding significance to promote public health.

## 2. Methods

### 2.1. Search Strategy

The Cochrane Library, MEDLINE, EMBASE, Science Citation Index, The China Journal Full-Text Database, Chinese Scientific Journals Full-Text Database, and Chinese Biomedical Literature Databases were searched from their earliest record to December 2014 with the terms (cranberr^*∗*^ or whortleberr^*∗*^ or bilberr^*∗*^ or lingonberr^*∗*^ or Blueberry Plant or Huckleberry Plant or* Vaccinium macrocarpon* or* Vaccinium myrtillus* or* Vaccinium vitis-idaea*), in combination with the medical subject headings. The related article function also was used to expand the search results. We did not restrict any languages during the searching. Hand searching was made by retrieving the reference lists of every obtained study for additional studies. Unpublished data were obtained through contacting authors. We identified ongoing trials by searching https://clinicaltrials.gov/, the UK National Research Register and Meta-Register of controlled trials on the Internet.

### 2.2. Study Selection

Randomized controlled clinical trials (irrespective of language, date of trial, blinding, or publication status) were included in meta-analysis as long as they were conducted in adult subjects with a duration equal to or over two weeks and contained a true control group. Trials only with baseline and after treatment values for synthesizing risk (mean) differences were included. The outcome measures were differences of serum total cholesterol (TC), HDL-C, LDL-C, and triglycerides (TG) between postrandomization baselines and after treatments. Eligible interventions were capsules of single isolated component or mixtures of different kinds of anthocyanins from* Vaccinium berries*. Interventions in forms of diets were also included as long as they compared* Vaccinium* berries containing treatments with* Vaccinium* berries depleting controls. Trials were excluded from meta-analysis if data required for pooling were missing (i.e., baseline mean and standard deviation [SD], end mean and SD, or change by group) or if studies involved children or pregnant participants or patients with conditions that required cholesterol-lowering medical treatment.

### 2.3. Data Extraction and Quality Assessment

All abstracts identified by the above search strategies were assessed for subject relevance. The full text of all relevant abstracts was downloaded from databases and meticulously assessed for inclusion. Data abstraction form was introduced to record details of study design, participants, setting and timing, interventions, patient characteristics, and outcomes. Data abstraction was strictly performed independently by two reviewers, with disagreement solved by discussion with the third researcher.

All studies that met the selection criteria were assessed for methodological quality to determine the risk of bias for each outcome. Two reviewers independently assessed the risk of bias according to the criteria stated in the Cochrane Collaboration Handbook [26], with disagreements resolved by discussion with the third researcher. The following methodological domains were considered: sequence generation, allocation concealment, blinding of participants, incomplete outcome data, selective outcome reporting, and other potential risk factors.

### 2.4. Statistical Analysis

We conducted the meta-analysis to determine the effect of* Vaccinium* berries on TC, HDL-C, LDL-C, and TG after summarizing available data from all trials reporting results. Blood lipid levels were unified in mmol/L. If cholesterol levels (TC, HDL, and LDL) or triglyceride levels were published in mg/dL, amounts were multiplied by a factor of 0.02586 for cholesterol and 0.0113 for triglycerides to convert to mmol/L. Results for continuous outcomes were expressed as weighted mean difference. All statistical analyses were performed with Review Manager (RevMan version 5.1.6) [27] by inputting the number of participants and the means and SDs of lipid concentrations at endpoint in the two comparison groups. For groups with four treatment arms, we grouped together all the experimental groups and compared them with the control group, respectively [28].

Chi-squared statistic and *I* ^2^ statistic were used to assess heterogeneity between trials and the extent of inconsistency apart. If there was a significant heterogeneity, a random-effects statistical model was introduced to confirm the summary results. A fixed-effect model was also applied to merge case estimates and their 95% CIs, unless there was a significant heterogeneity. Subgroup analysis was introduced by* Vaccinium* subclasses to explore obvious therapeutic differences among trials. Sensitivity analyses were also performed by removing one study at a time to assess any impact of study quality on the effect estimates.

## 3. Results

### 3.1. Trial Flow

**Figure 1:**
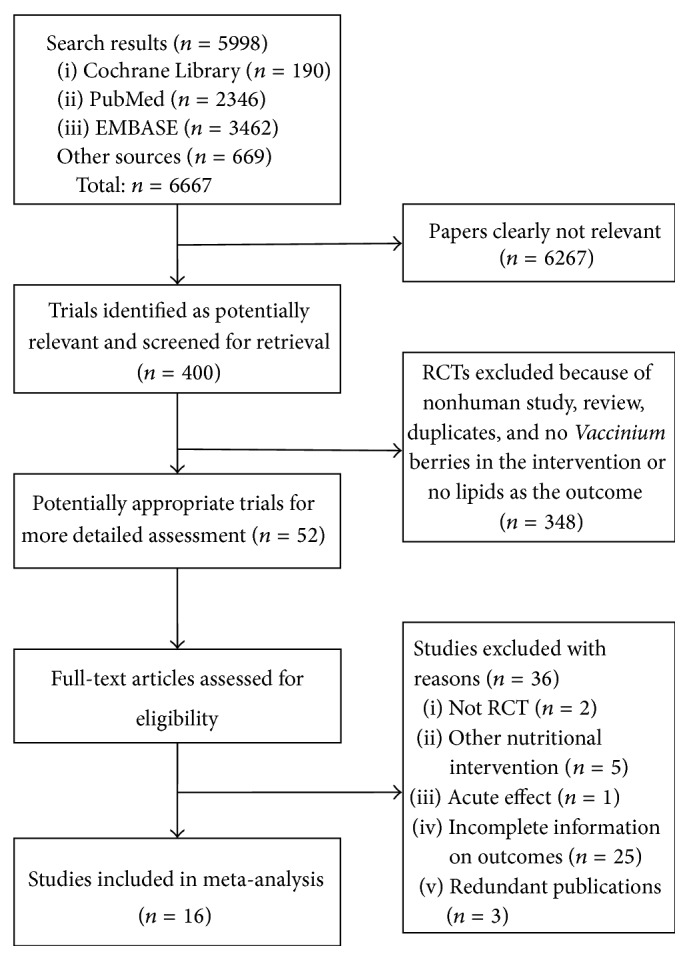
Flow diagram of trial selection.

From Figure 1 we can see the flowchart studies from the initial results of publication searches to the final inclusion. Sixteen trials of* Vaccinium* berries versus control for serum lipids with 19 comparison arms including 1109 patients were recruited in this meta-analysis. Reasons for exclusion mostly were nonrandomization, lack of control, insufficient original data, or baseline values.

### 3.2. Study Characteristics

Characteristics of each trial were given in Table 1. The population being studied were adults with or without some chronic diseases. Means of interventions varied from berry juice to capsules containing berry extracts. Cranberry is introduced in 7 trials, blueberry in 3 trials, bilberry in 4 trials, and whortleberry in 2 trials. The average intake of anthocyanins was up to 742 mg and length of treatment was ranging from 2 to 24 weeks. Three trials recruited healthy subjects while thirteen included participants with cardiovascular risk factors. Two studies just recruited female, one recruited male, and ten recruited subjects with both genders.

### 3.3. Risk of Bias in Included Studies

**Figure 2:**
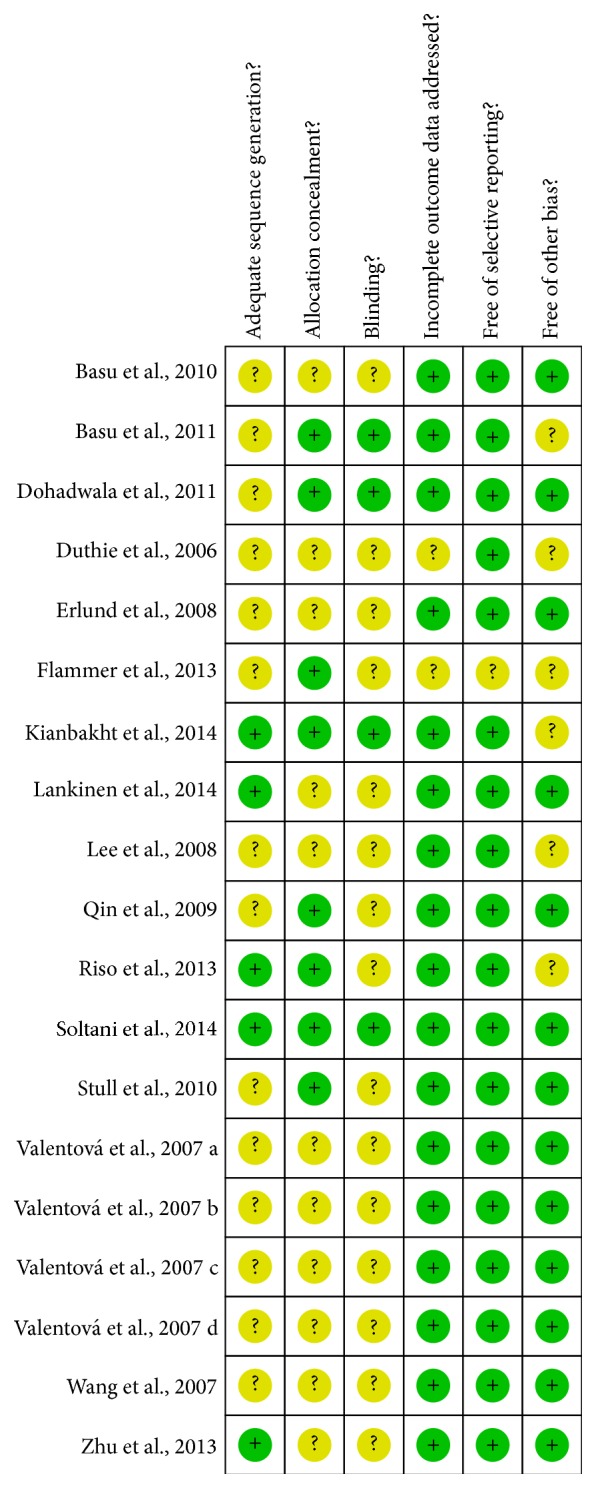
Methodological quality of included studies.

The assessment of risk of bias is presented in Figure 2. All sixteen trials were claimed as randomized, but only five trails clearly described how randomization was achieved. The attempts to mask participants and researchers were reported in 5 studies and 4 studies, respectively, but none of the trials reported masking the outcome assessors. Allocation concealment was clearly adequate in 8 trials. None of the trials carried out ITT analysis. The dropout rates for the trials ranged from 0 to 27.3%. We considered two trials [37, 41] to have unclear risk of bias for this domain, as we could not determine whether the high dropout of more than 20% could have affected the treatment estimates.

For other potential sources of bias, we focused on two aspects, namely, baseline comparability and the financial support on trials. The intervention and control groups in all trials were reported or appeared to be comparable at baseline for the lipid levels. Seven trials reported that the studies received financial support from nonprofitable organization such as university research grant.

### 3.4. Effects of Interventions

#### 3.4.1. Outcome: Total Cholesterol

**Figure 3:**
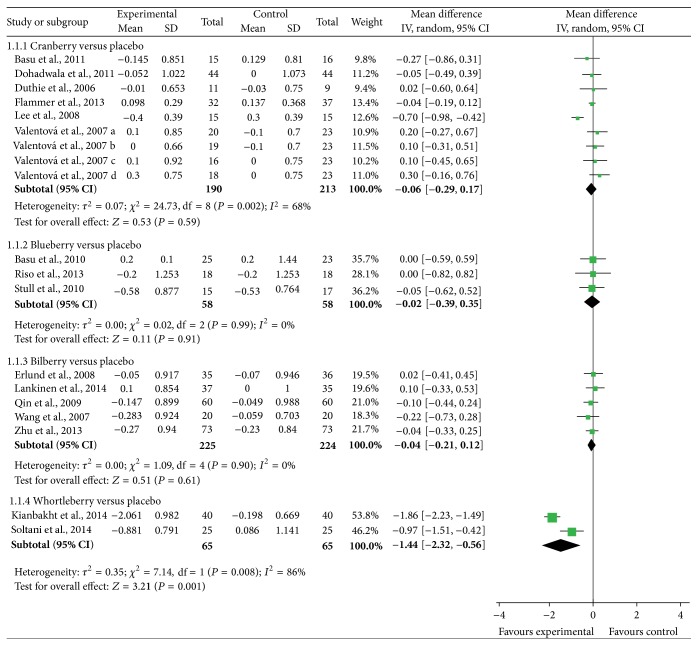
Forest plot of comparisons of* Vaccinium* berries versus control (outcome: total cholesterol).

Figure 3 shows no significant differences between intervention and control groups in total cholesterol were found for comparisons between cranberry, blueberry, bilberry, and controls. However, two trials [41, 42] that compared whortleberry with placebo show significant differences between the treatments favouring whortleberry (mean difference = −1.44 (95% CI: −2.32, −0.56) mmol/L; *P* = 0.001).

Sensitivity analyses revealed that the heterogeneity of included studies in cranberry group on total cholesterol was highly affected by the study performed by Lee et al. When this study was removed from the analysis, the heterogeneity changed from 68% to 0%. However, it showed no significant difference on the total effect in the cranberry group.

#### 3.4.2. HDL-C

**Figure 4:**
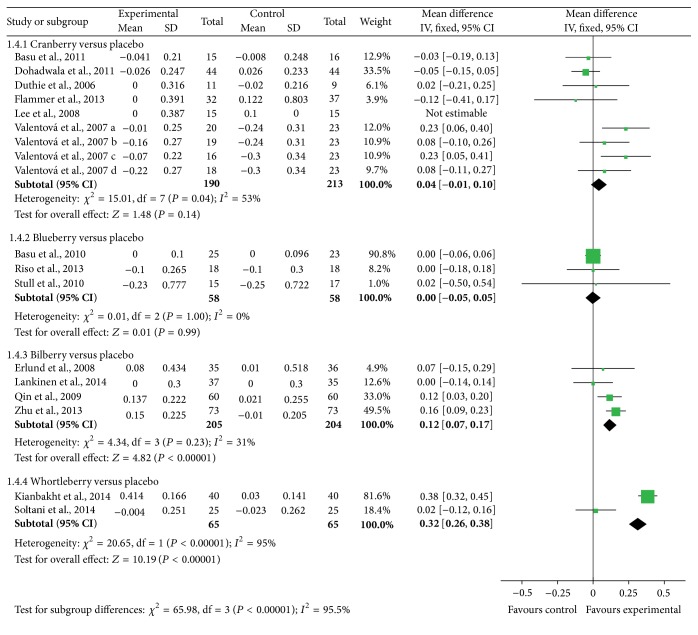
Forest plot of comparisons of* Vaccinium* berries versus control (outcome: HDL-C).

Significant differences were found in HDL-C among studies in bilberry groups (mean difference = 0.12 (95% CI: 0.07, 0.17) mmol/L; *P* < 0.001) and whortleberry groups (mean difference = 0.32 (95% CI: 0.26, 0.38) mmol/L; *P* < 0.001) while no obvious differences were observed in HDL-C levels among cranberry and blueberry groups (shown in Figure 4).

#### 3.4.3. LDL-C

**Figure 5:**
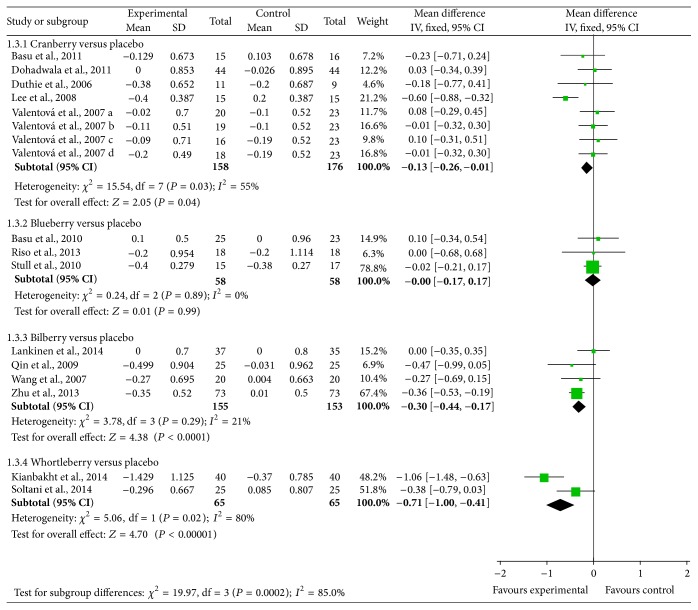
Forest plot of comparisons of* Vaccinium* berries versus control (outcome: LDL-C).

Statistical differences were found in comparisons of* Vaccinium* berries versus control in LDL-C levels (mean difference = −0.20 (95% CI: −0.28, −0.12); *P* < 0.001). Particularly, bilberry (mean difference = −0.30 (95% CI: −0.44, −0.17) mmol/L; *P* < 0.001) and whortleberry (mean difference = −0.71 (95% CI: −1.00, −0.41) mmol/L; *P* < 0.001) groups show more benefit comparing with other treatments. Changes are also observed in cranberry groups (mean difference = −0.13 (95% CI: −0.26, −0.01) mmol/L; *P* = 0.04). However, results pooled for three placebo-controlled trails in blueberry groups show no significant differences between intervention and control groups (shown in Figure 5).

Sensitivity analysis revealed that the heterogeneity of included studies in cranberry group on LDL-C was highly affected by the study performed by Lee et al. When this study was removed from the analysis, the heterogeneity changed from 55% to 0%. It showed significant difference on the total effect in the cranberry group (*P* value changed from 0.04 to 0.99). Considering the study performed by Lee et al. is of medium quality and does not match the exclusion standard, we can only suggest that cranberry may have some effect on reducing LDL-C.

#### 3.4.4. Triglycerides

**Figure 6:**
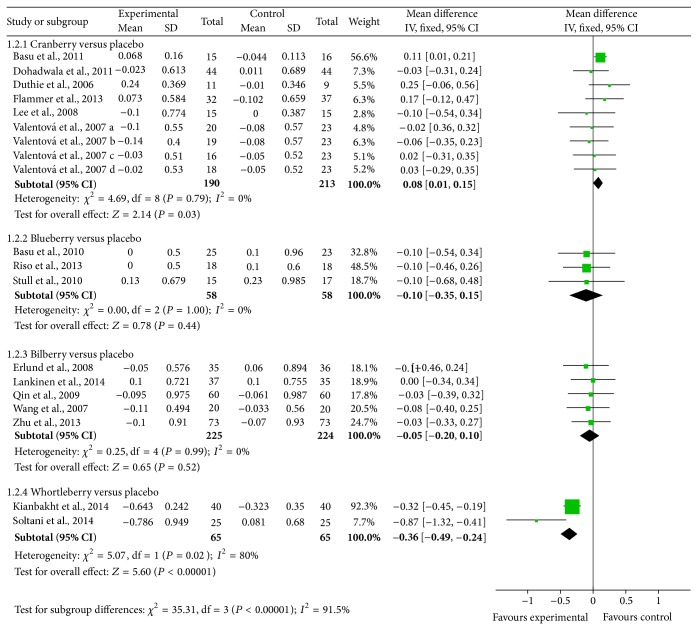
Forest plot of comparisons of* Vaccinium* berries versus control (outcome: triglycerides).

No significant differences were found in TG between groups for all comparisons except for the comparisons between whortleberry and control groups (mean difference = −0.36 (95% CI: −0.49, −0.24)) (shown in Figure 6).

#### 3.4.5. Side Effects

Two trials [32, 33] reported side effects of nausea or dyspepsia in a small number of participants in the intervention groups (1 and 2 people, resp.). Basu et al. [37] reported a dropout of 27% in intervention group due to nausea, vomiting, constipation, or diarrhea. However, the number was appreciably similar to that in placebo groups (28% dropouts due to personal reasons). Four trials stated no healthy complaints of participants. Four trials [25, 28, 35, 42] investigated the biomarkers of hepatic and renal functions or hematology. All reported no changes in liver function, biochemistry, or hematology. The rest of nine trials did not have adequate information about side effects.

#### 3.4.6. Publication Bias

**Figure 7:**
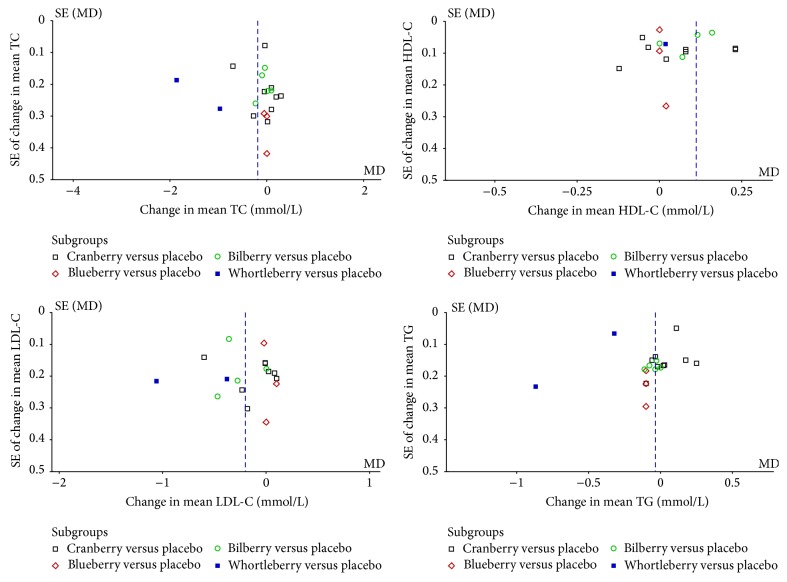
Funnel plots of trials included in the meta-analysis on the effect of* Vaccinium* berries on TC, HDL-C, LDL-C, and TG.

Funnel plots use Begg's test [43] of trials to investigate the effect of* Vaccinium* berries on cholesterol (TC, LDL, and HDL) and triglyceride levels, indicating no publication bias except for total cholesterol (shown in Figure 7).

## 4. Discussion

### 4.1. Main Summary of Findings

Sixteen RCTs with nineteen comparison arms involving 1109 patients were included in this review. The findings from two trials [41, 42] clearly show that whortleberry is significantly superior to placebo in lipid reduction, decreasing the TC, TG, and LDL-C and increasing HDL-C at the same time. However, given that the *I* ^2^ values were high (86% in TC, 80% in TG, 80% in LDL-C, and 95% in HDL-C), the results should be interpreted with caution. Differences in the daily doses and sources of anthocyanins, age of subjects, and lipids baseline values as well as the different durations of the trials might contribute to some extent to the observed statistical heterogeneity. Besides, bilberry groups show significant differences in reducing LDL-C and increasing HDL-C in comparison with other treatments. The lipids-lowering properties of anthocyanins have been linked to the inhibition of cholesteryl ester transfer protein and the suppression of LDL oxidation, as well as improvement in HDL-associated paraoxonase 1 activity [15, 25, 39, 44]. For many other* Vaccinium* subclasses or other comparison groups, there was insufficient evidence to draw conclusions about efficacy.

Valentová et al. [28] compared dried cranberry juice with placebo in lipids reducing effects in two different anthocyanins doses (400 mg/d and 1200 mg/d) and two different durations (4 weeks and 8 weeks). However, no significant changes were found in four comparison arms, showing no dose-response and time-response effects. These may be due to the poor absorption of anthocyanins. Various berry (but nor cranberry) anthocyanin glycosides have been found to be absorbed and excreted into urine unmetabolized by both human beings and animals. Only 0.1% of the amount ingested was excreted into the urine [45]. Ohnishi et al. [46] recently found that cranberry anthocyanins are excreted into urine at a total amount of 5% of the dose consumed within 24 h with a maximum excretion period between 3 and 6 h after consumption. Additional speculation is that the most abundant active material may not necessarily produce the highest concentrations of biologically active ingredients. Future studies should focus on the acute effect of anthocyanins, trying to find its clinical relevant endpoint. Besides, as for bilberry and whortleberry, we need to explore the dose-dependent effect of anthocyanins and verify whether synergistic effects are necessary with some other nutrients.

### 4.2. Strengths and Limitations

The importance of anthocyanins as a part of heart healthy diet has been widely proved. Purified anthocyanins mixture reduced the inflammatory response in hypercholesterolemic subjects [39] and consumption of the wild blueberry drink for 6 weeks significantly reduced the levels of oxidized DNA bases and increased the resistance to oxidatively induced DNA damage [35]. Epidemiology studies support the protective effect of cranberry and blueberry on urinary tract infection [47–49]. As far as we know, this report is the first systematic review assessing the effectiveness of the range of* Vaccinium* subclasses rich in anthocyanins on lipid improvement within RCTs. This systematic review, the most comprehensive to date, includes a quantitative pooling of results and assessment of risk of bias of included studies.

The shortcomings of the sixteen trials are represented in Figure 2. Several trials failed to contain adequate methodological information, such as method of randomization, allocation concealment, blinding, funding, and dropouts, which are essential for assessing risk of bias. In conclusion, the included studies have moderate risk of bias. In addition, since purified anthocyanins extracted from berries used by Qin et al. [25] and Zhu et al. [39] and berry diets in other researches both show the lipids reducing effect, it is not clear whether the anthocyanins themselves (rather than other bioactive substances) are solely or partially responsible for the observed effects, since the specific biological active ingredients mediating the lipids improvement of the berries belonging to the* Vaccinium* genus are not yet characterized. In addition, even though we have undertaken extensive searches for published material, we still could not exclude the possibility that studies with negative findings remain unpublished.

## 5. Conclusion

### 5.1. Implications for Practice

Results from this review provide some evidence of the beneficial effects of bilberry and whortleberry on lipids reduction. However, recommending bilberry and whortleberry for lowering lipids levels is not justifiable on current evidence because of the limited data. As objects in whortleberry group are diagnosed hyperlipidemia patients whereas not all of the objects in other groups exhibit dyslipidemia, we cannot draw the conclusion that other types of* Vaccinium* berries have no effect on lipids lowering. In addition, although whortleberry can reduce serum lipids, it cannot always lower the lipids to the normal level as statins do, so it can be just recommended as an adjunct instead of replacement. More studies are needed before these berries can be widely recommended for cardiovascular health. Anyway, adding some berries in our daily diets is good for human health.

### 5.2. Suggestion to Future Trails

The included trials were all small and had methodological problems. Further trials should be designed rigorously with large sample sizes to confirm the effectiveness of* Vaccinium* berries for lipids improvement. Besides, further researches need to assess dose-response effects, be of adequate duration, and report all primary outcomes. Additionally, anthocyanins are commonly consumed as part of a normal diet, and a future focus on purified anthocyanins extracted from different subclasses of* Vaccinium* is needed to determine their specific lipids-lowering effects.

## Figures and Tables

**Table 1:** Characteristics of the 16 included studies.

Trials	Country	Subject	Design	Sample size (I/C dropouts)	Cranberry form	Dosage of anthocyanins	Control	Length of study
Duthie et al. 2006 [29]	UK	Healthy subjects	Parallel	11/9 (0)	Cranberry juice	2.1 mg/d	Placebo	2 w

Valentová et al. 2007 [28]	Czech Republic	Healthy women	Parallel	21/20/16 (8)	Dried cranberry juice	2.6 mg/d and 7.8 mg/d	Placebo	4 w and 8 w

Wang et al. 2007 [30]	Taiwan	Healthy subjects	Parallel	20/20	Cranberry vinegar	Not stated	Placebo	10 w

Lee et al. 2008 [31]	Taiwan China	Type 2 diabetes	Parallel	15/15 (0)	Cranberry capsule	Not stated	Placebo	12 w

Basu et al. 2011 [32]	USA	Women with metabolic syndrome	Parallel	15/16 (5)	Cranberry juice	24.8 mg/d	Placebo	8 w

Dohadwala et al. 2011 [33]	USA	Coronary artery disease	Crossover	44 (3)	Cranberry	94 mg/d	Placebo	4 w

Flammer et al. 2013 [34]	USA	Cardiovascular risk factors	Parallel	32/37 (15)	Cranberry juice cocktail	69.5 mg/d	Placebo	8 w

Riso et al. 2013 [35]	Italy	Men with cardiovascular risk factors	Crossover	18 (2)	Blueberry drink	375 mg	Placebo	6 w

Stull et al. 2010 [36]	USA	Obese, insulin-resistant	Parallel	15/17	Blueberry smoothie	668 mg/d	Placebo	6 w

Basu et al. 2010 [37]	USA	Obese, metabolic syndrome	Parallel	25/23 (18)	Blueberry beverage	742 mg/d	Placebo	8 w

Erlund et al. 2008 [38]	USA	Cardiovascular risk factors	Parallel	35/36 (1)	Bilberry, lingonberry	299 mg/d	Placebo	8 w

Qin et al. 2009 [25]	China	Dyslipidemic subjects	Parallel	60/60	Bilberry, blackcurrant	320 mg/d	Placebo	12 w

Zhu et al. 2013 [39]	China	Hypercholesterolemia	Parallel	73/73 (4)	Bilberry, blackcurrant	320 mg/d	Placebo	24 w

Lankinen et al. 2014 [40]	Finland	Metabolic syndrome	Parallel	37/34	Bilberry whole grain, fish	Not stated	Whole grain	12 w

Kianbakht et al. 2014 [41]	Iran	Hyperlipidemia	Parallel	40/40 (25)	Whortleberry	7.35 mg/d	Placebo	8 w

Soltani et al. 2014 [42]	Iran	Hyperlipidemia	Parallel	25/25 (4)	Whortleberry	90 mg/d	Placebo	4 w

I: intervention group; C: control group.
